# Is *Vibrio fluvialis* Emerging As a Pathogen with Epidemic Potential in Coastal Region of Eastern India Following Cyclone *Aila*?

**DOI:** 10.3329/jhpn.v28i4.6036

**Published:** 2010-08

**Authors:** Subhajit Bhattacharjee, Sayantani Bhattacharjee, Baishali Bal, Reshmi Pal, Swapan Kumar Niyogi, Kamalesh Sarkar

**Affiliations:** ^1^ Tagore Society for Rural Development, Rangabelia, South 24 Parganas, India; ^2^ National Institute of Cholera & Enteric Diseases, P-33 CIT Road, Scheme XM, Kolkata 700 010, India

**Keywords:** Diarrhoea, Cyclone, Disasters, Natural, *Vibrio cholerae*, *Vibrio fluvialis*, India

## Abstract

An isolated area with diarrhoea epidemic was explored at Pakhirala village of the Sundarbans, a coastal region of South 24 Parganas district of West Bengal, eastern India. The Pakhirala village was surrounded by other villages affected by a similar epidemic. The affected villages experienced this epidemic following the cyclone *Aila*, which had hit the coastal region of the Sundarbans in eastern India. In Pakhirala, the situation was the worst. Within a span of six weeks (5 June–20 July 2009), 3,529 (91.2%) of 3,871 residents were affected by watery diarrhoea. Of all the cases (n=3,529), 918 (26%) were affected by moderate to severe diarrhoea. In other villages, 28,550 (70%) of the 40,786 people were affected; of them, 3,997 (14%) had moderate to severe watery diarrhoea. The attack rate and the severity of the cases were significantly higher in Pakhirala village compared to other affected villages. The laboratory results revealed that *Vibrio fluvialis* was the predominant pathogen in Pakhirala village (5 of 6 laboratory-confirmed organisms) whereas *Vibrio cholerae* O1 Ogawa was the predominant pathogen in other villages of Gosaba block (7 of 9 bacteriologically-confirmed organisms). This result indicates that *V. fluvialis* behaves more aggressively than *V. cholerae* O1 in an epidemic situation with a higher attack rate and a different clinical picture. An in-depth study is required to explore its pathogenicity in detail, geographical distribution, and possible control measures, including development of specific vaccine preparation and determination of its efficacy.

## INTRODUCTION

On 25 May 2009, a major cyclone named *Aila* at a speed of 120-140 km per hour hit the coastal islands of the Sunderbans, the largest delta islands in the world, situated in the southern part of West Bengal, eastern India. The Sunderbans has been facing the brunt of ‘global warming’ recently because of which the islands have been reported to be under water during the high tides. During the cyclone, a high tidal wave of about eight metres in height resulted in the destruction of the river embankments for a distance of around 150 km. This brought about massive devastation of man and property in these areas.

*Aila* hit the islands of the Sundarbans leading to flood that caused an epidemic of watery diarrhoea all over these islands just a week after the calamity struck this area. This epidemic was preceded by a focal outbreak of watery diarrhoea (with or without presence of blood in stool) caused by *Vibrio fluvialis* in February 2009 in Pakhirala village of Gosaba block in the Sundarbans. A team from the National Institute of Cholera & Enteric Diseases, Kolkata, carried out the investigation and confirmed the aetiological agent at that time. The organism was detected from the piped water-supply system used for drinking purpose in the affected village and from stool samples of infected patients. Except the treatment of the affected cases, no specific intervention, particularly provision of environmental sanitation, was offered to the community. Fortunately, there was a natural decline of the outbreak within six weeks from its onset. This rose the question whether *V. fluvialis* could lead to an epidemic in coastal regions, particularly following natural disasters, such as flood which is common in these delta areas. An investigation was made to find out the outbreak-causing potentiality and epidemiology of *V. fluvialis* in the Sundarbans following *Aila*.

## MATERIALS AND METHODS

### Study area

**Fig. 1.**
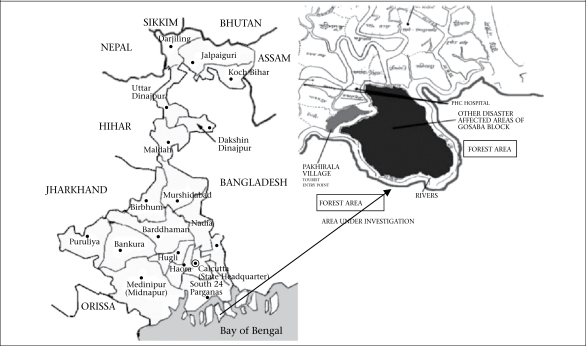
Map of West Bengal showing Pakhirala village and other disaster-affected villages of Gosaba block, Sundarbans area, 5 June–20 July 2009

Villages across the Gosaba block of the Sundarbans were affected during the post-*Aila* diarrhoea epidemic. Pakhirala village was one of the many affected villages (Fig. 1).

One of the major attractions of this village is that tourism in the Sundarbans is based on this village. Of the 44,657 inhabitants in 80 villages of Gosaba block of the Sundarbans, 71% were reported to be affected. All patients with features of moderate to severe watery diarrhoea were attended and/or admitted to the nearby health facilities that included primary or block health centres and private hospitals run by non-governmental organizations (NGOs). Admission registers of these health establishments were consulted to understand the epidemiology of the outbreak.

### Study population and sample collection

For the purpose of this study, ‘diarrhoea cases’ were defined as those who passed three or more loose or liquid stools per day, or more frequently than is normal for the individual (1). Distinction among ‘mild’, ‘moderate’, and ‘severe’ diarrhoea was based on degree of dehydration. Patients with no signs or symptoms of dehydration were regarded as suffering from mild diarrhoea. Patients were considered to be suffering from ‘moderate diarrhoea’ if they showed the signs of sunken eyes, dry mouth, increased thirst, restlessness or irritable behaviour, and slow retraction of skin-pinch (1,2). Patients were regarded as suffering from severe diarrhoea if signs of severe dehydration were present, such as drowsiness or unconsciousness, inability to drink, weak and rapid radial pulse, low/undetectable blood pressure, cool, moist extremities, and lack of urine output (1,2).

A subset (n=100) of the affected people of Pakhirala village was interviewed using a pretested questionnaire and examined clinically to understand their morbidity profile. Following this, stool samples/rectal swabs were collected in Cary-Blair media from the affected people of this village and from other villages. At Pakhirala village, stool samples/rectal swabs were collected from patients visiting an NGO-managed private hospital. Since this was the only healthcare facility in the region, the affected people from different parts of the village came here for treatment.

Samples were collected from the eligible and willing patients after obtaining verbal consent. In the case of drowsy patients and children, consent was obtained from the accompanying adults and guardians. Stool samples were not collected from patients belonging to the same household to ensure broader geographic distribution of the cases. Addresses of the patients were also verified from the hospital registers to ensure that they came from different parts of the village and were not clustered in the same neighbourhood. Care was taken to collect specimens before the start of antibiotics. Stool samples/rectal swabs were collected in a similar way from patients visiting primary or block health centres at the other affected villages. Samples were transported to the laboratory of the National Institute of Cholera & Enteric Diseases (NICED), Kolkata, a World Health Organization reference laboratory for cholera. However, due to lack of facilities, only 37 stool samples (10 from the affected people of Pakhirala village and 27 from the people of other affected villages, such as Parasmani, Rangabelia, Dayapur, Johar Colony, Sadhupur, and Shantigachi) reached the laboratory on time for bacteriological confirmation.

Of the 10 affected people from Pakhirala village, four were aged less than 10 years, three were aged 20-40 years, and three were aged over 40 years; four were male, and six were female. Of the 27 affected people from other villages, three were aged less than 10 years, six were aged 10-20 years, 10 were aged 21-40 years, and eight were aged over 40 years; 15 were male.

### Laboratory procedure

Rectal swabs collected from the diarrhoeal patients were inoculated into alkaline peptone water [1% bacto peptone (Difco), 1% NaCl, pH 8.5] and incubated overnight. One loopful of the enriched sample was plated onto thiosulphate citrate bile salts sucrose (TCBS) agar (Eiken Chemical Co. Ltd., Tokyo, Japan), followed by incubation at 37 °C overnight. Yellow colonies on the TCBS plates were tested for oxidase reaction. Oxidase-positive strains that did not agglutinate with *Vibrio cholerae*-specific antisera (O1 and O139) were further characterized biochemically in the API 20E identification system (bioMérieux). Salt tolerance was determined by growth of strains at 37 °C in 1% peptone broth supplemented with 7% NaCl but not in the absence of NaCl. The string test was performed using 0.5% sodium deoxycholate solution with fresh cultures grown on nutrient agar. The presence of other common enteric pathogens was also examined by standard procedures (3).

## RESULTS

In Pakhirala village, the situation was the worst. Within a span of six weeks (5 June–20 July 2009), 3,529 (91.2%) of the 3,871 residents were affected by watery diarrhoea. Of all the cases (n=3,529), 918 (26%) were affected by moderate to severe diarrhoea. In other villages, 28,550 (70%) of the 40,786 people were affected. Of those affected, 3,997 (14%) had moderate to severe watery diarrhoea. The attack rate and the severity of the cases were significantly higher (p<0.05) in Pakhirala village compared to other affected villages (Table 1). The laboratory results revealed that *V. fluvialis* was a predominant pathogen in Pakhirala village (5 of 6 laboratory-confirmed organisms was *V. fluvialis*, 1 was *Escherichia coli*) whereas *V. cholerae* O1 Ogawa was the predominant pathogen in other villages of Gosaba block (7 of 9 laboratory-confirmed organisms were found to be *V. cholerae* O1 Ogawa, and two were *E. coli*).

**Table 1.** Attack rate and moderate to severe case rate of diarrhoeal diseases among residents of Pakhirala village and surrounding areas of Gosaba block, Sunderban area, 5 June–20 July 2009

Village	Total population	All cases	Attack rate (%)
Pakhirala village	3,871	3,529	91
Other villages	40,786	28,550	70
Village	All cases	Moderate-severe cases	Moderate to severe case rate (%)
Pakhirala village	3,529	918	26
Other villages	28,550	3,997	14

**Fig. 2.**
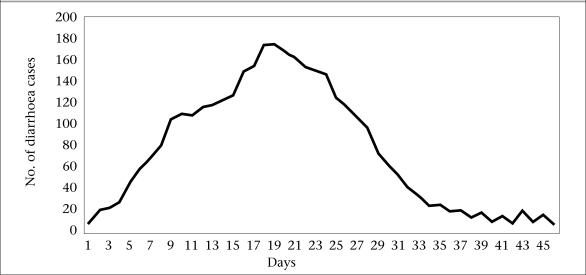
Epidemic curve showing moderate to severe diarrhoea cases reported to local health facilites of Pakhirala village, Gosaba block, Sundarbans area, 5 June–20 July 2009

Figure 2 shows the day-wise distribution of moderate to severe cases at Pakhirala village (n=918). The epidemic curve appears to be that of a common source epidemic with its peak around 18^th^ and 19^th^ days, followed by gradual decline of cases over the next 2-3-week period.

**Fig. 3.**
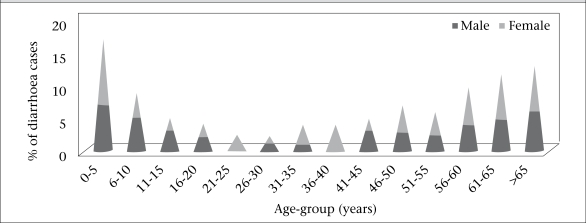
Age and sex distribution of reported moderate to severe cases (n=100) at Pakhirala village, Gosaba block, Sundarbans area, 5 June–20 July 2009

Figure 3 shows age and sex distribution of the cases in Pakhirala village. The disease was distributed mostly in extremes of ages. Males and females appeared to be equally susceptible in all age-groups, except in the age-groups of 20-25 years and 35-40 years, where all the affected were female.

Clinically, watery stool was the commonest presentation with or without presence of blood. Sixty-two percent of ill subjects had blood in their stools. Another important feature was abdominal pain, which was experienced by 57% of the participants (Table 2).

**Table 2.** Symptoms experienced by moderate to severe cases (n=100) at Pakhirala village, Gosaba block, Sundarbans area, 5 June–20 July 2009

Symptoms of disease due to *Vibrio fluvialis*	%
Watery stool	86
Blood in stool	62
Chills	23
Abdominal pain	57
Nausea	29
Vomiting	41
Muscle pain	12
Headache	7
Fever	21

## DISCUSSION

Several villages of Gosaba block of the Sundarbans were hit by the post-*Aila* flood that led to an epidemic of watery diarrhoea. The epidemic experienced by the people of Pakhirala village appeared to be different from that of the remaining affected villages. Epidemiologically, the attack rate in Pakhirala village was significantly higher compared to the remaining affected villages (91% vs 70%; p<0.05). The proportion of moderate to severe cases was also significantly higher in Pakhirala village compared to other villages (26% vs 14%; p<0.05). This indicates that the organism causing epidemic at Pakhirala village appeared to be more virulent. Clinically, most study participants suffered from watery diarrhoea. Other major symptoms were presence of blood in stool, abdominal pain, and vomiting, and only a few experienced mild fever. These symptoms were similar to other *Fluvialis*-related diarrhoea cases as observed in Jakarta, Indonesia, and Bangladesh (4,5). A striking feature reported in the present study is that 62% of the ill subjects had blood in their stools. Gastroenteritis caused by *V. fluvialis* often leads to presence of erythrocytes/blood in stool (6). Notably, the combination of watery diarrhoea with or without blood in stool and abdominal pain was not observed in patients of other affected villages. This also supports the fact that the epidemic in Pakhirala village was different from the other villages. *V. fluvialis* is generally common in infants, children, and young adults (4,6,7). In the present study, children aged ≤10 years and elderly people aged 55 years or above were more affected than others. As reported in a study (4), the cases were equally distributed between both the sexes.

The laboratory-confirmed report supports the view that the predominant pathogen causing epidemic at Pakhirala village was *V. fluvialis* whereas epidemic in the remaining villages was dominated by *V. cholerae* O1 Ogawa. Circumstantial evidence also provides support in favour of a focal outbreak of watery diarrhoea which preceeded this post-*Aila* epidemic in February 2009. During this outbreak, confirmed *V. fluvialis* cases were reported by the laboratory of National Institute of Cholera & Enteric Diseases, Kolkata (unpublished data). The organism was detected from the drinking-water sources (piped water system) and from stool samples of the infected patients at that time. Prior presence of *V. fluvialis* in Pakhirala village environment has probably facilitated a different kind of epidemic not experienced by other affected villages. A similar kind of outbreak was never reported from any other village of Gosaba block.

*Fluvialis* cases were treated with injection ceftriaxone. Antibiotic sensitivity revealed that the organism was sensitive to doxycycline, norfloxacin, second/third-generation cephlosporin and azithromycin. It was, however, resistant to ciprofloxacin, nalidixic acid, and ampicillin. A safe and effective vaccine will probably prevent the indiscriminate use of antibiotics and, thus, prevent the development of resistant and more virulent strains (8). This is specially significant in resource-poor settings, such as the Sundarbans where medical facilities are hardly available leading to the indiscriminate use of antibiotics.

Furniss *et al*. documented the first diarrhoea case caused by *V. fluvialis* in 1977 (9). It was later described and named by Lee *et al*. in 1981 (10). Subsequently, sporadic cases of gastroenterities and outbreaks of diarrhoea were reported from different parts of the world (11–17). The largest-known outbreak due to *Fluvialis* during October 1976–June 1977 was reported by Huq *et al*. in Bangladesh (5). One small epidemic due to foodborne *Fluvialis* was reported from Maharashtra, India, in 1981 (18). However, no waterborne epidemic caused by *V. fluvialis* has so far been reported from this region.

This is perhaps the first report of an epidemic of watery diarrhoea caused by *V. fluvialis* in a southeast Asian country, following a natural disaster like *Aila*. Its epidemic potential and higher pathogenicity compared to *V. cholerae* is of great concern. It is also interesting to observe an epidemic caused predominantly by *V. fluvialis* in one village and *V. cholerae* in other villages of the same district block (Gosaba). The existence of *V. fluvialis* in Pakhirala village is probably facilitated by costal region (saline water) since the pathogen is known to be halophilic and is generally found in marine and estuarine environments (19–22).

Surprisingly, although the villages other than Pakhirala village are also surrounded by brakish sea-water, the outbreak due to *V. fluvialis* has not affected these villages. Further investigation is, thus, perhaps required to determine the epidemiology of *V. fluvialis* in this epidemic. If *V. fluvialis* has an epidemic potential similar to *V. cholerae* O139, it might cause a disaster in future. Experience suggests that *V. cholerae* O139 Bengal, which first emerged during 1992-1993 along the coastal lines, later caused large epidemics of cholera in Bangladesh, India, and neighbouring countries (23,24). The situation in Pakhirala is further complicated by the presence of tourism which allows a large number of national and insternational tourists to gather each year. An urgent community-based intervention with safe environmental sanitation is, therefore, necessary to prevent similar epidemics in fututre in the affected areas. Much interest has recently been shown by the scientific community on development of a safe and immunogenic cholera vaccine to be used in cholera-endemic areas.

In India, so far, the most common pathogen causing epidemics of cholera has been *V. cholerae* O1 (25–27). Most vaccines developed so far target *V. cholerae* O1 and O139 (28). If in future, *V. fluvialis* gains importance as an epidemic-causing *Vibrio*, especially in the costal areas, question arises whether blanket coverage by present vaccines will be effective in reducing epidemics of diarrhoea. Question also remains whether the present vaccines will provide any cross-immunity against *V. fluvialis*-associated infection. Another matter of concern is that blanket coverage by cholera vaccines can lead to a false sense of security in areas more prone to infection due to *V. fluvialis*. If *V. fluvialis*-related epidemics become widespread in future, perhaps more specific vaccines need to be developed. Strategies can be thought of to immunize people in the coastal areas before the beginning of the rainy season to protect them from flood-related diarrhoeal diseases.

### Limitations

We acknowledge that, due to heavy flood, a difficult working situation, and scarcity of resources, it was not possible for us to collect an adequate number of stool samples/rectal swabs for laboratory confirmation. Even of those samples collected, only a few reached the laboratory on time for proper bacteriological diagnosis. Although confirmatory tests were carried out to diagnose the causative agent, due to lack of resources, it was not possible to carry out other molecular tests, such as pulsed-field gel electrophoresis. We, therefore, report the findings of this study based on circumstantial evidence and clinical findings which showed clear demarcation between cases from Pakhirala village and those from other villages. Cases from the former region presented with watery diarrhoea with or without the presence of blood whereas those from other villages presented with only watery diarrhoea and no trace of blood.

### Conclusions

The Sundarbans, the world's largest delta islands that harbours the lushness of the Mangrove forests, has been labelled a World Heritage site by the United Nations Educational, Scientific and Cultural Organization and has been short-listed as one of the new Seven Wonders of the world. Pakhirala, the gateway to domestic and foreign tourists visiting the Sundarbans, has only one tourist accommodation. Every year, a considerable number of tourists visit this village and are exposed to its contaminated drinking-water. The situation, thereby, poses a threat and can lead to the spread of an epidemic caused by *V. fluvialis*. With no alternative water supply and existence of an ideal environmental condition suitable for the survival of *V. fluvialis*, an epidemic of a magnanimous scale due to diarrhoea can result in future. Moreover, the islands of the Sundarbans are often affected by flood, and as such, *V. fluvialis* can very soon find its way to other islands, thereby affecting the wider geographic area. The situation can become worse if *V. fluvialis* does indeed have an epidemic potential higher than that of *V. cholerae* O1 as was found in this study. Further studies are required to better understand the epidemiology of *V. fluvialis*, especially as epidemic-causing bacteria. The need of the hour, thus, lies in the realization of the present situation and calls for necessary steps of precaution without which an epidemic of a huge scale perhaps remains impending in the near future. The cluster of islands already on the verge of depletion, thus, calls for help to escape a looming disaster in the future.
